# Magnetic Properties and Microstructure of FePt(BN, X, C) (X = Ag, Re) Films

**DOI:** 10.3390/nano13030539

**Published:** 2023-01-29

**Authors:** Jai-Lin Tsai, Chun-Yu Sun, Jhih-Hong Lin, Yi-Yuan Huang, He-Ting Tsai

**Affiliations:** 1 Department of Materials Science and Engineering, National Chung Hsing University, Taichung 402, Taiwan; 2 Instrument Center, The Office of Research & Development, National Chung Hsing University, Taichung 402, Taiwan

**Keywords:** out-of-plane coercivity, perpendicular magnetocrystalline anisotropy, spin–orbital coupling, columnar grains, granular structure

## Abstract

A sputtered FePt(BN, Re, C) film, here boron nitride (BN), was compared to a reference sample FePt(BN, Ag, C). Intrinsically, these films illustrate a high anisotropy field (H_k_) and perpendicular magnetocrystalline anisotropy (K_u_),although the reference sample shows a higher value (H_k_ = 69.5 kOe, K_u_ = 1.74 × 10^7^ erg/cm^3^) than the FePt(BN, Re, C) film (H_k_ = 66.9 kOe, K_u_ = 1.46 × 10^7^ erg/cm^3^). However, the small difference in the anisotropy constant (K2/K1) ratio presents a close tendency in the angular dependence of the switching field. Extrinsically, the out-of-plane coercivity for the reference sample is 32 kOe, which is also higher than the FePt(BN, Re, C) film (H_c_ = 27 kOe), and both films present lower remanence (Mr_(parallel)_/Mr_(perpendicular)_ = 0.08~0.12), that is, the index for perpendicular magnetic anisotropy. The higher perpendicular magnetization for both films was due to highly (001) textured FePt films, which was also evidenced by the tight rocking width of 4.1°/3.0° for (001)/(002) X-ray diffraction peaks, respectively, and high-enough ordering degree. The reference sample was measured to have a higher ordering degree (S = 0.84) than FePt(BN, Re, C) (S = 0.63). As a result, the Ag segregant shows stronger ability to promote the ordering of the FePt film; however, the FePt(BN, Re, C) film still has comparable magnetic properties without Ag doping. From the surface and elemental composition analysis, the metallic Re atoms found in the FePt lattice result in a strong spin–orbital coupling between transition metal Fe (3d electron) and heavy metals (Re, Pt) (5d electron) and we conducted high magnetocrystalline anisotropy (K_u_). Above is the explanation that the lower-ordered FePt(BN, Re, C) film still has high-enough Ku and out-of-plane Hc. Regarding the microstructure, both the reference sample and FePt(BN, Re, C) show granular structure and columnar grains, and the respective average grain size and distributions are 6.60 nm (12.5%) and 11.2 nm (15.9%). The average widths of the grain boundaries and the aspect ratio of the columnar grain height are 2.05 nm, 1.00 nm, 2.35 nm, and 1.70 nm, respectively.

## 1. Introduction

Energy-assisted magnetic recording is an alternative way to overcome the trilemma effect (superparamagnetic effect of CoCrPt oxides) in conventional perpendicular magnetic recording (PMR) and heat-assisted magnetic recording (HAMR) with L10 FePt media is the mainstream technology to target the hard disk area density beyond 4 Tb/in2 in the near future. The L10 FePt film, which has higher magnetocrystalline anisotropy (Ku), is the optimal media in the HAMR system [1,2,3,4] and the hard disk drive (HDD) based on FePt media is currently being tested on data center customers [5,6,7,8].

To apply FePt media to HAMR, many works have discussed this extensively for decades. For HAMR media, perpendicular magnetic anisotropy (PMA) of the FePt film was a key requirement to extend the area density to ultrahigh and increase the capacity in HDD [1,2,3,4]. After annealing the disordered face-centered cubic FePt alloy with nearly equal atomic compositional range, the ordered L10 phase with face-centered tetragonal (fct) structure, which has high Ku [1,2,3,4], was formed. As a result, magnetron sputtering and high-temperature deposition became the main technologies to prepare ordered L10 FePt films with high Ku. Many elements, for example, silver (Ag), copper (Cu), etc., have been doped to lower the L10 FePt ordering temperature and Curie temperature, which influenced the process deposition temperature and the laser writing (heat) power during thin-film engineering [9,10,11]. In addition, the structure requirement for perpendicular magnetic anisotropy tuned the surface energy and lattice misfit strain between the FePt and MgO-based underlayer [12,13,14,15,16] and CrRu seed layers [14,17,18], and sputtered the FePt film with (00L) orientation. In addition, to obtain a uniform granular structure and columnar grains to avoid noise and increase the signal-to-noise ratio, multiple segregants are required and Ag, carbon (C), and boron nitride (BN) are typical grain boundary materials for perpendicular FePt media [5,6,19]. The C has strong phase separation and diffusive ability to separate FePt grains and will initiate lateral growth and interrupt the columnar grains when the C concentration is much higher than the solubility. The amorphous BN was hard enough to support the FePt grains’ growth upward in the columnar morphology. The Ag was used to enhance the FePt ordering via diffusion of Ag, which induces atom replacement through vacancies [5,6,9,19,20]. To study the FePt-X composite films, interface chemistry in heterostructures [21] and other functional alloy, oxide and composite films prepared in sputtering [22,23] and other thin-film formation technologies [24,25] was also addressed.

Multiple segregants are required in heat-assisted perpendicular magnetic recording media and BN, Ag, and C are necessary materials to co-sputter with the FePt layer and form a typical microstructure, which shows granular structure and columnar grains. In this work, the Ag segregant was replaced by the 5d element rhenium (Re). According to the reference report, 5d elements have large spin–orbital coupling that will influence the magnetocrystalline anisotropy of 3d metals; for example, in the order/disorder FePt alloy, the 5d element significantly enhanced the magnetocrystalline anisotropy through spin–orbital coupling and 3d–5d hybridization [26,27]. Based on this argument, different Re 5d elements were doped in the FePt system to form an FePt(Re) alloy film, and the grain boundary materials were parts of BN and C. Originally, the Ag was added to improve the FePt ordering degree, which is proportional to the magnetic anisotropy, and we then tried to break this relationship by preparing FePt(BN, Re, C), which has a lower ordering degree but comparable Ku. The achievement of this work is adding Re to replace the Ag segregant in the reference sample and that the FePt(BN, Re, C) film presents comparative magnetic properties and microstructures.

Furthermore, the magnetic media noise was reduced by reducing the intrinsic- and extrinsic parts of the switching field distribution (SFD) [2,3,27,28] and the SFD of two samples was measured. The major and minor magnetic hysteresis loops were measured to define and understand the magnetic characteristics, for example, dispersed magnetic H_k_, misaligned c-axis, non-uniform grain size in extrinsic parts [26,27], and the grains’ long-term dipole and short-term exchange coupling from extrinsic contribution [2,3].

## 2. Materials and Methods

The FePt(BN, Ag, C) and FePt(BN, Re, C) films were deposited by direct current magnetron sputtering on MgO (100) single-crystal substrate. The sputtering deposition equipment was designed to have main and pre-chambers and the substrate was moved via the load-lock feedthrough to the substrate holder, which has a heating system formed of a halogen lamp (OSRAM, 1000W) and cathodes (AJA, A320, North Scituate, MA, USA) set up in the main chamber. The two-inch-diameter composite targets FePt(BN, Ag, C) and FePt(BN, Re, C) were used. In order to compare the elemental Ag and Re doping effects precisely, the MgO (100) single crystal was used to provide the optimal baseline for the epitaxial growth of (001) textured FePt film. The MgO (100) substrate (HF-Kejing, 10 mm × 10 mm × 0.5 mm) was heated at 470 °C (the substrate surface temperature) for dry cleaning and then the magnetic layers with thickness of 15 nm were sputtered under an Argon working pressure of 10 mTorr at 470 oC by the composite targets. The deposition rate of FePt(BN, Ag, C) (15 nm) and FePt(BN, Re, C) (15 nm) was 0.0372 nm/s and 0.0421 nm/s, respectively. In this study, the FePt(BN, Ag, C) was named as the reference sample because of the typical segregants (BN, Ag, C) used in HAMR media.

The crystal structure was measured using standard X-ray diffraction (XRD) (BRUKER, D8 Discover). The magnetization curves with in-plane and out-of-plane measured hysteresis loops were performed with a superconducting quantum interference device (SQUID) magnetometer (MPMS-XL). The sample microstructure was observed using transmission electron microscopy (TEM, JEOL JEM-2010). The surface analysis was measured using X-ray photoelectron spectroscopy (XPS, ULVAC-PHI 5000).

## 3. Results and Discussion

**Figure 1:**
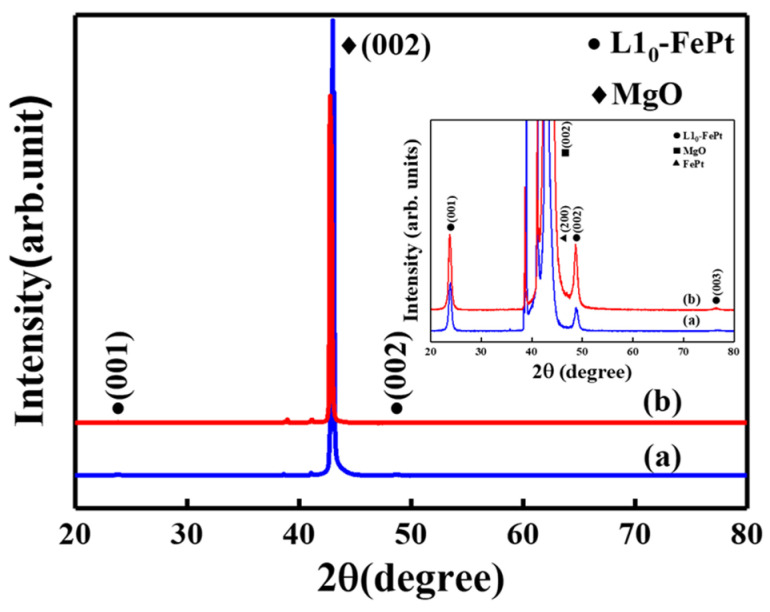

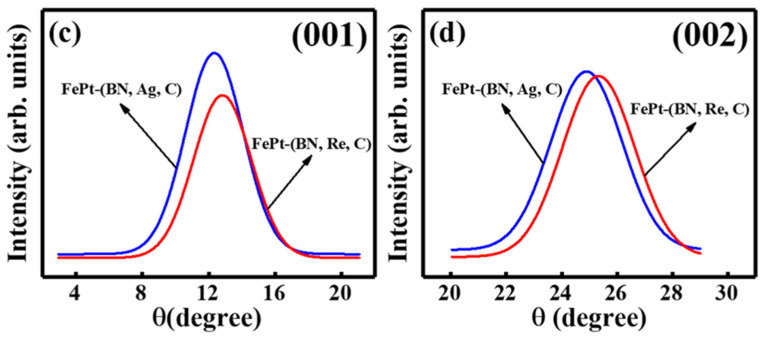
XRD patterns of (**a**) reference sample, FePt(BN, Ag, C), and (**b**) FePt(BN, Re, C) film. In the inset picture, the MgO (100) single-crystal peak was trimmed from top to observe the ordered L1_0_ FePt (00L) diffraction peaks. FePt(001)/(002) rocking curves of (**c**) FePt(BN, Ag, C) and (**d**) FePt(BN, Re, C) films (the fcc FePt (JCPDS card no. 00-029-0718), the tetragonal (ordered L10 FePt [JCPDS 43-1359])).

Figure 1 shows XRD patterns of (a) FePt(BN, Ag, C) (reference sample) and (b) FePt(BN, Re, C) films. Based on the crystal structure, the strong (001) and weak (003) superlattice peaks were present because of the ordered L10 FePt phase (JCPDS 43-1359) and the (002) fundamental reflection peak was also indexed (fcc FePt, JCPDS card no. 00-029-0718). The lattice constant “c” was calculated using the (001) diffraction peaks and the values are 0.3728 nm and 0.3743 nm, respectively. In addition, the (110) diffraction peak was measured using grazing incident diffraction (GID) that is similar to the in-plane XRD and the lattice constant “a” that was estimated with values of 0.3889 nm and 0.3857. The reference sample presents a smaller c/a ratio (0.959), which means higher ordering (more tensile stress on axis “a” and compressive force on axis “c”) as compared to the FePt(BN, Re, C) film with c/a= 0.970. Another way to estimate the FePt ordering degree is by using B. E. Warren’s equation [29,30]; the ordering degree is (I_(001)_/I(_002)_)^1/2^/(I*_(002)_/I*_(001)_)^1/2^ and the values are 0.84 and 0.63, respectively. The (I*_(002)_/I*_(001)_)^1/2^ value was calculated using film thickness and the width of half intensity in rocking curve, in theory [29,30], and I_(001)_I_(002)_ is the peak’s integrated intensity ratio for textured (001) FePt films and the respective values are 2.04 and 1.15 in (a) and (b). In Figure 1b, there is the fct/fcc structured FePt (200) tiny peak, indexed at 47.0°, which contributes to the c-axis misalignment and lowers the ordering degree of the FePt(BN, Re, C) film. The c-axis misalignment mentioned above can be measured using the rocking curves and width at a half intensity of FePt (001)/(002); the peaks are 4.09°/3.03° and 4.09°/3.07° in Figure 1c,d. The (002) rocking curve was asymmetric due to the overlap of (200) and (002) peaks and a little wider inthe FePt(BN, Re, C) sample. In summary, the reference sample and FePt(BN, Re, C) film were hetero-epitaxially grown on MgO(100) and have a strong (001) texture.

**Figure 2:**
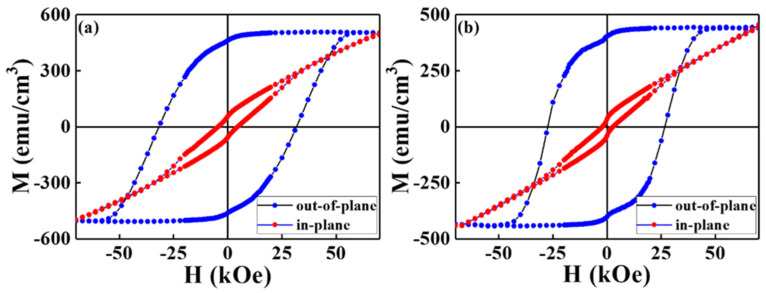
Room-temperature magnetic hysteresis loops of (**a**) reference sample, FePt(BN, Ag, C), and (**b**) FePt(BN, Re, C) films measured in easy (out-of-plane to film surface)- and hard (in-plane to film surface)-axis directions.

The perpendicular Hk magnetic anisotropy of the reference sample and FePt(BN, Re, C) film was proved by the in-plane and out-of-plane magnetic hysteresis loop measurements. The easy magnetization curve (out-of-plane) shows full and huge hysteresis and the hard direction is a linear-like curve with small loop areas in Figure 2. The out-of-plane coercivity (Hc) is 32 kOe, 27 kOe, and the (Mr_(in-plane)_)/Mr_(out-of-plane)_ ratio, which qualifies the perpendicular anisotropy as 0.12, 0.08, and the negative nucleation field, which is defined at 0.95 Ms, is −2.5 kOe and −1.8 kOe, respectively. In Table 1, the reference sample illustrates a higher magnetic anisotropic field (Hk), which equals to 2Ku/Ms (Hk = 69.5 kOe), magneto-crystalline anisotropy (Ku= 1.74 × 10^7^ erg/cm^3^) and saturation of magnetization (Ms = 502 kG), than the FePt(BN, Re, C) film intrinsically. The magnetic anisotropy field Hk was defined as the intersection point of easy- and hard-axes magnetic hysteresis loops. The reference sample FePt(BN, Ag, C) film shows a higher ordering degree (S) and also has higher magnetic anisotropy (Ku than the FePt(BN, Re, C) film. Only 63% of the FePt(BN, Re, C) grains are ordered but this film still has high-enough Ku and Hc. For reference, the 5% concentration Co-Re shows higher Ku than Co-Pt [26]. It is suggested that Fe-Re also contributes to extra Ku magnetic anisotropy due to the higher spin-orbital coupling of heavy metal Re and hybridization with 3d Fe electrons. As a result, the high magnetic anisotropy (Ku and out-of-plane Hc were proved in the relative lower ordered (S = 0.63) FePt(BN, Re, C) film without Ag segregant.

**Figure 3:**
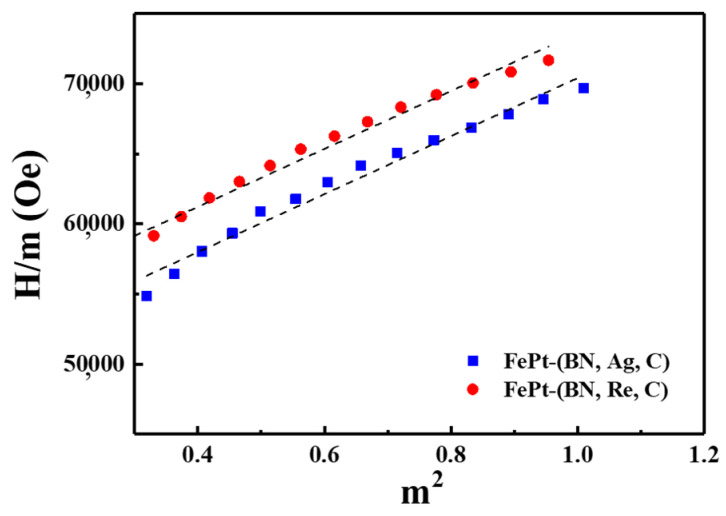
Analysis of the hard-axis magnetization curve based on Sucksmith–Thompson method for reference FePt(BN, Ag, C) and FePt(BN, Re, C) films measured at room temperature.

From Table 1, the respective anisotropy fields (HK) of the reference FePt(BN, Ag, C) and FePt(BN, Re, C) films are 69.5 and 66.9 kOe, and it is noteworthy that the values of Ku were often estimated from the slopes on the magnetization curves measured using a maximum applied field lower than the magnetic anisotropy Hk of L10-FePt, which was likely to result in certain errors in the estimation of Ku and which might confuse the discussion. Figure 3 illustrates the individual linear part in hard-axis curves for the reference sample, and the FePt(BN, Re, C) film and the K1 and a high-order anisotropy constant K2 were estimated using the following equation, respectively, [31,32]

(1) $$\frac{H}{m}=\frac{2K_{1}^{\prime }}{\mu_{0}M_{2}}+\frac{4K_{2}}{\mu_{0}M_{s}}m^{2}$$

where *m* = *M*/*M*_s_ is the normalized magnetization and $K_{1}^{\prime }$ is the second-order anisotropy constant (Sucksmith–Thompson method). From the H/*m* versus *m*^2^ plot, the *K*_1_ and *K*_2_ constants were conducted by the interception and the slope of the fitting curve [31,32]. The *K*_1_ values are closer for the reference sample (*K*_1_ = 1.18 × 10^7^ erg/cm^3^) and FePt(BN, Re, C) (*K*_1_ = 1.16 × 10^7^ erg/cm^3^) after fitting with the Sucksmith–Thompson method and the *K*_2_ values are 3.06 × 10^6^ and 2.59 × 10^6^ erg/cm^3^, respectively. The *K*_2_/*K*_1_ ratios of reference and FePt(BN, Re, C) samples were 0.26 and 0.22 and the small change in the anisotropy constant ratio means a close tendency in the angular dependence of the switching field. 

The (dynamic) ordering temperature, degree, and magnetic anisotropic Ku constant were influenced by the FePt chemical composition, which was fixed to avoid any misleading. The MgO (100) single-crystal substrate was used and it is suggested that the structure variant of the FePt film was due to the slight dissolution of segregants at a high deposition temperature. The quite different ordering degree in the reference sample (S = 0.84) and FePt (Re, C, BN) film (S = 0.63) was because of the structure variant that also influenced the magnetic anisotropy Ku. Based on the reference reports [31,32], the K_u1_ and K_u2_ are proportional and independent to the ordering degree (S), respectively. In this study, the values of the ordering degree and magnetic anisotropic constant were a direct response to the Ag and Re segregants because the concentration of C and BN doping was the same in both samples. Adding the Ag element truly promotes the ordering degree and has higher magnetic anisotropic constant (K_u_). In the Sucksmith–Thompson method, the *K*_1_, which determines the switching behavior, has close values for two samples (d*K*_1_ ~ 0.02) and the *K*_2_, which dominates the thermal stability, has large differences (d*K*_2_ ~ 0.47). The *K*_1_ is proportional to the ordering degree but the FePt(Re, C, BN) film presents extra spin–orbital coupling between Fe and Pt(Re), which enhances the *K*_1_ value. As a result, the FePt(BN, Re, C) film shows a lower *K*_2_/*K*_1_ value (0.22 < 0.26).

**Figure 4:**
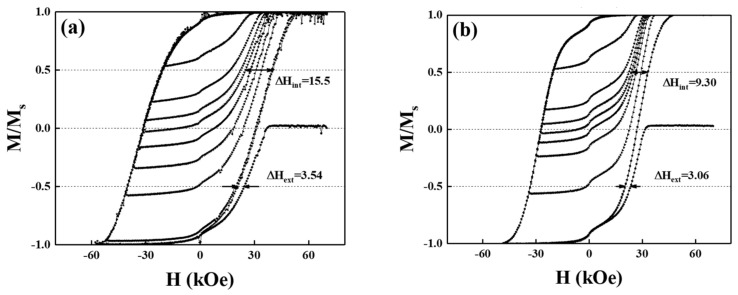
Major and minor loops of (**a**) reference FePt(BN, Ag, C) film and (**b**) FePt(BN, Re, C) film. The minor loop is used to measure intrinsic and extrinsic switching field distribution (SFD).

Figure 4 presents the major and minor (recoil in variant field) magnetization loops to study the relationships between the magnetic switching behavior and grains’ morphology, and the intrinsic and extrinsic switching field distribution was analyzed. When the loops recoiled in the coercivity field, half of the grains (50%) were switched in the easy axial direction and the parameters ΔH_int_ and ΔH_ext_ were collected after comparison with the major magnetic hysteresis curves. For reference FePt(BN, Ag, C) and FePt(BN, Re, C) films, the intrinsic (ΔH_int_ = 15.5 kOe, 9.30 kOe) and extrinsic (ΔH_ext_ = 3.54 kOe, 3.06 kOe) switching field distributions were determined in Figure 4a,b, respectively.

The intrinsic switching field distribution comes from grain-size distribution (σv_olume_), magnetic anisotropy field dispersion (σ_Hk_), and misalignment of the c-axis (σ_axis_), and the extrinsic SFD comes from the grains’ dipolar and exchange coupling [2,3,27,28]. The standard deviation (σ_int_) of intrinsic SFD is estimated from the ΔHint/1.35 [2,3,27,28], and the (σ_int_)2 can be written as the summary of (σ_volume_)^2^ + (σ_axis_)^2^ + (σ_Hk_)^2^, because of the close values in rocking width (Δθ_50_) and average grain size in both samples; the grain-size distribution (σ_volume_) and misalignment of the c-axis (σ_axis_) terms were dropped. As a result, the SFD can be evaluated by the magnetic anisotropy field dispersion (σ_Hk_) and the FePt (BN, Re, C) film illustrates smaller values. The small difference in extrinsic SFD (ΔH_ext_) means that the equal grain decoupling in granular structure for two samples and the long-range magnetostatic coupling and inter-granular coupling were not considered in this study.

**Figure 5:**
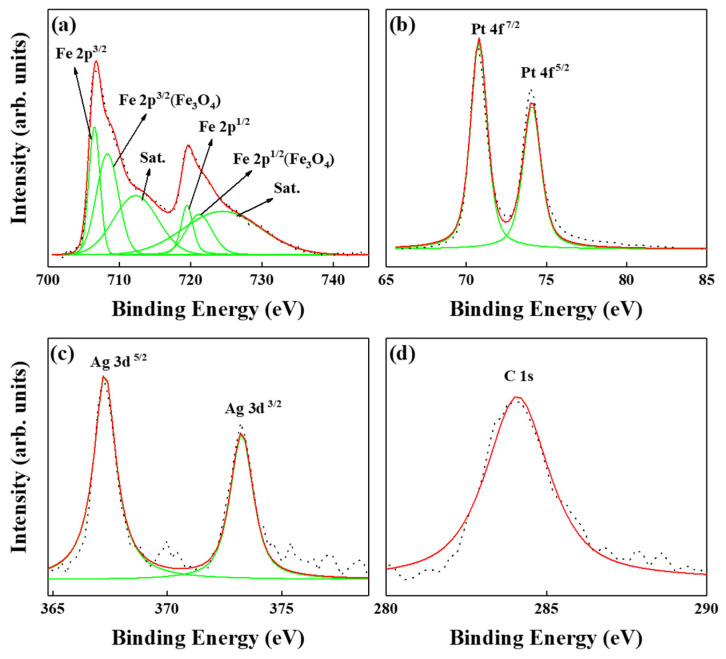
XPS of reference FePt(BN, Ag, C) film, (**a**) the metallic Fe, iron oxide was indexed, (**b**) Pt, (**c**) Ag, and (**d**) C.

**Figure 6:**
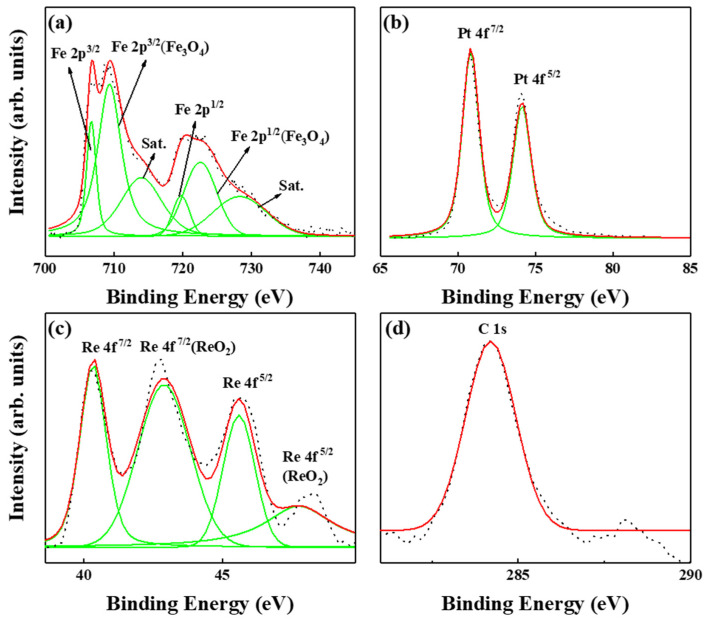
XPS of FePt(BN, Re, C) film, (**a**) metallic Fe, iron oxide were indexed, and (**b**) Pt, (**c**) metallic Re, and ReO_2_ were indexed and (**d**) C.

Figure 5 and Figure 6 present the XPS of FePt(BN, Ag, C) and FePt(BN, Re, C) films. In Figure 5, in spite of metallic Fe, Pt, C, and Ag peaks, ferrite Fe_3_O_4_ and Fe satellite peak (Sat.) were also detected from the film surface after peak resolution. From the spectra line shape shown in Figure 5 and Figure 6a, there are distinctive shoulders at the binding energy around 710 eV and 720 eV, and the Fe 2p3/2 and 2p1/2 core-level binding energy was considered and the Fe_3_O_4_ phase was observed both in FePt(BN, Ag, C) and FePt(BN, Re, C) films. The magnetite Fe_3_O_4_ (ferrimagnet) is a soft magnetic material and responds to the slight kink illustrated in the out-of-plane magnetization curves at zero field in Figure 2a [33,34].

The ferrite Fe_3_O_4_ was also observed in the XPS of the FePt(BN, Re, C) film in Figure 6a and the kink in the magnetization curve was more clear in Figure 2b. For inverse spinel ferrite Fe_3_O_4_, the Fe^3+^ ions are divided into tetrahedral (A) and octahedral (B) sites equally and the Fe^2+^ ions are in B sites. In Figure 5 and Figure 6, the Fe^2+^/Fe^3+^ ratio is higher in the FePt(BN, Re, C) film, which means that larger soft-magnetic moments contributed to the surface of the hard magnetic layer and a clear kink was observed in the hysteresis loops, as evidenced in Figure 2b [35,36,37]. In addition, the metallic Re, and ReO_2_ appeared after considering the Re 4f^5/2^ and 4f^7/2^ core-level binding energy in Figure 6c. According to reference [38], the ReO2 is dark blue or black in color and the crystal structure is in the monoclinic form below, 300 °C, and orthorhombic between 300 and 1500 °C. The FePt(BN, Ag, C) and FePt(BN, Re, C) thin-film surface oxidations were both indexed in XPS and the oxides were soft-magnetic Fe_3_O_4_ and ReO_2_, which may cause the lower ordering degree in the FePt(BN, Re, C) film due to the deviation in near-equal atomic FePt. Actually, the soft ferrite Fe_3_O_4_ causes the kink in magnetic hysteresis loops shown in Figure 2a,b and needs to be avoided in practical applications.

**Figure 7:**
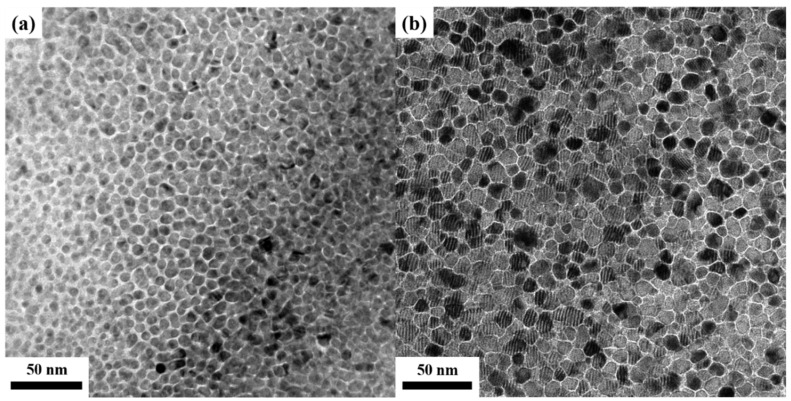

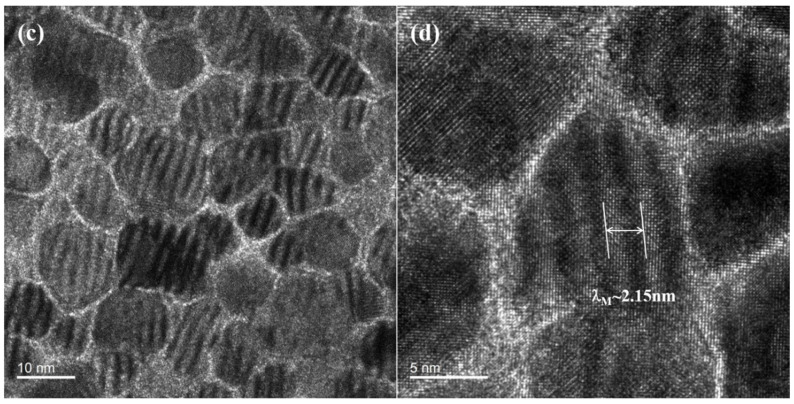
Plane-view TEM images of (**a**) FePt(BN, Ag, C) and (**b**) FePt(BN, Re, C) films; (**c**) Moire fringes in FePt(BN, Re, C) film and (**d**) selected grain to estimate the periodicity of Moire fringe.

**Figure 8:**
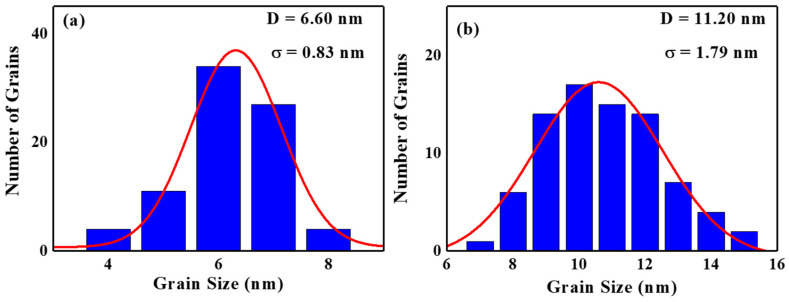
Average grain-size distribution <D> and standard deviation (σ) of (**a**) reference FePt(BN, Ag, C) sample and (**b**) FePt(BN, Re, C) films.

From the plane-view TEM images, the FePt(BN, Ag, C) and FePt(BN, Re, C) films show a typical uniform granular structure in which the FePt grains were separated by the segregants (Figure 7). In Figure 8a,b, the FePt grains are mono-distributed with average grain sizes <D> of 6.60 nm and 11.2 nm, and the grain-size distributions, defined by (σ: standard deviation)/<D>, are 12.6% and 16.0% in the reference sample and FePt(BN, Re, C) film, respectively. The reference sample shows smaller and more uniform grains than the FePt(BN, Re, C) film and reflects the higher nucleation field (HN) that was defined at the field of 0.95 Ms and out-of-plane coercivity (H_c_), as presented in Table 1. However, the grains’ density in the FePt(BN, Re, C) film was higher than in the reference sample because of the smaller grain boundaries’ width (1.00 nm < 2.05 nm), which was estimated by the subtraction of <D_c_>(centre-to-centre average grain size) to <D> (average grain size). 

The coherence between FePt and MgO(100) was confirmed by the orientation and periodicity of the bright-field Moire fringes caused by the interference of diffraction spot patterns between FePt and MgO and the periodicity (*λ*) is given by Equation (2) [39]

(2) $$\lambda =\frac{1}{\left|\Delta g\right|}=\frac{d_{FePt}d_{MgO}}{\sqrt{d_{FePt}^{2}+d_{MgO}^{2}-2d_{FePt}d_{MgO}\cos\alpha }}$$

where *α* is the relative angle between FePt(001) and MgO(002), and *d*_FePt_ and *d*_MgO_ are spacing between planes, ∆g vector is reciprocal vectors between *g*_FePt_ and *g*_MgO_ [39].

The mixed Moire fringe patterns can be observed in Figure 7c and the average periodicity is 2.33 nm (63.5°); for example, the individual period pattern is 2.15 nm, as measured in Figure 7d. When the epitaxial coherency is perfect, the alpha value is 0 but the rotational angle between FePt(001) and MgO(002) in this work is around 63.5o, calculated by Equation (1), which means non-perfect epitaxial growth in the FePt(BN, Re, C)/MgO system. 

**Figure 9:**
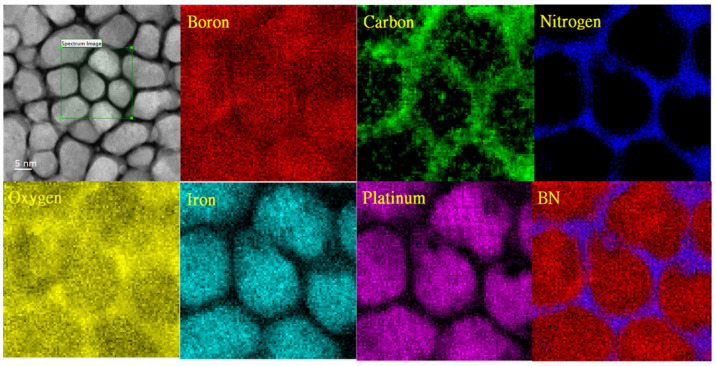
EELS mapping of Fe, Pt, B, N, C, and O elements by HRTEM for FePt(BN, Re, C) film.

For the light atoms, for example, B, C, and N elements we chose to detect them via electron energy loss spectroscopy (EELS) with beam sizes smaller than 5 nm; the EELS spectrum of FePt(BN, Re, C) is shown in Figure 9. The B element was observed both in FePt lattice and grain boundaries and the C and N elements were segregated in the grain boundaries. After overlapping, part of the BN and C was found clearly in the grain boundaries. The unexpected O element, which may come from the natural oxidation during high-temperature deposition, was also observed at the grain’s boundary and the surface oxides, for example, ReOx and Fe3O4 were indexed in the XPS. 

**Figure 10:**
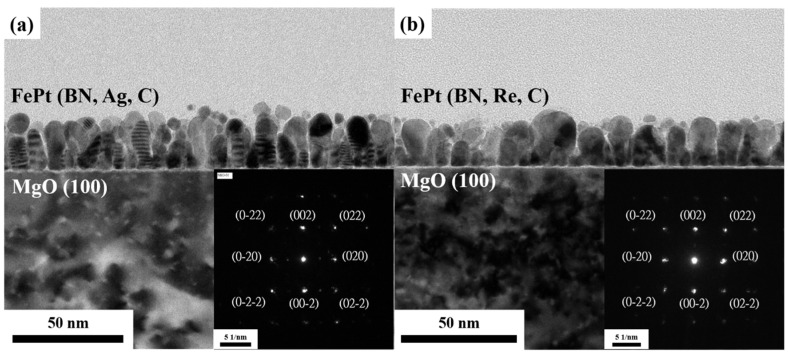
Cross-section TEM images of (**a**) FePt(BN, Ag, C) and (**b**) FePt(BN, Re, C) films. Insets are nanobeam diffraction patterns.

The columnar grain structure was observed both in the reference sample and FePt(BN, Re, C) film in Figure 10a,b and the average aspect ratio of grains was defined from the film thickness to grain width (t/w), where the values are 2.35 and 1.70, respectively. Some columnar grains were interrupted and non-uniform due to the thicker FePt film (15 nm), but the highest-aspect-ratio FePt grains were demonstrated in this study. The insets show that the nanobeam diffraction patterns and the {0KL} planes were indexed.

**Figure 11:**
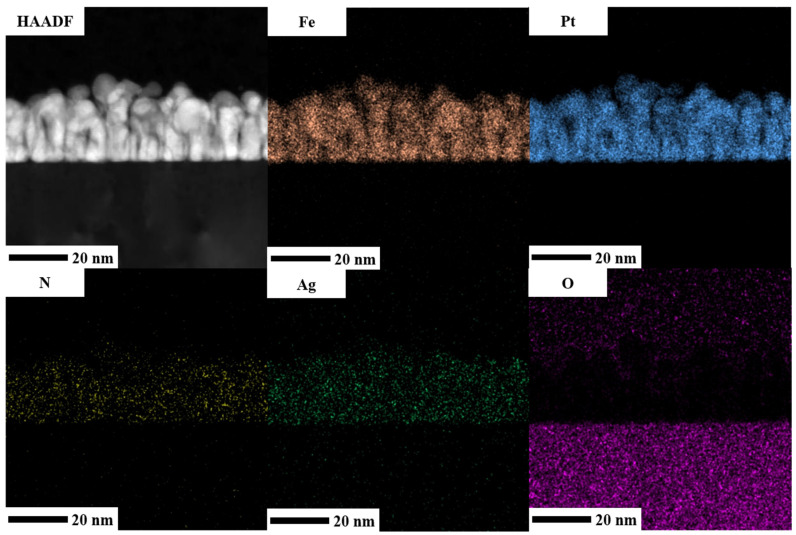
High-angle angular dark-field (HAADF) image and EDX mapping of Fe, Pt, Ag, N, and O elements of FePt(BN, Ag, C) film.

**Figure 12:**
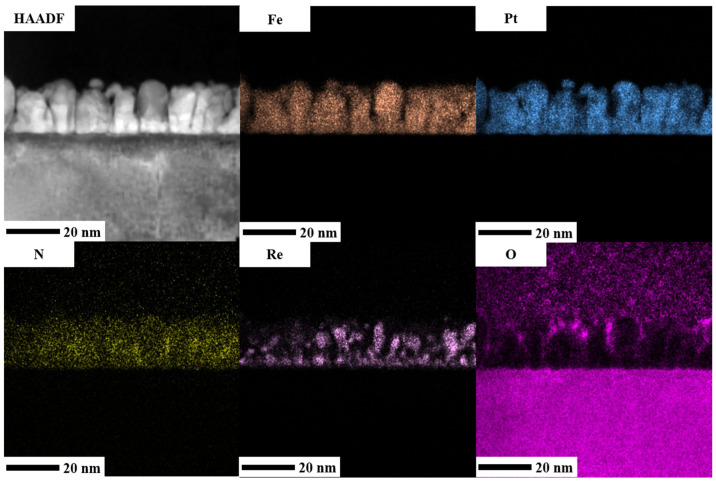
High-angle angular dark-field (HAADF) image and EDX mapping of Fe, Pt, N, and O elements of FePt(BN, Re, C) film.

To provide more evidence of the element distribution, energy-dispersive X-ray (EDX) mapping was performed to analyze the composition in the reference sample and FePt(BN, Re, C) film. The cross-sectional high-angle angular dark-field (HAADF) images are shown in Figure 11 and Figure 12. Both Fe and Pt elements are presented almost in the same position and grain areas and most of the Ag element were distributed in the FePt columnar grains’ edges, as shown in Figure 11. Because the Ag atoms were rejected or diffused out at a high deposition temperature and occupied the Fe sites that decreased the coordination number, a Ag shell was formed [1,40]. The signal of B and C elements was not observed in EDX mapping but proved in the EELS image. The N element was distributed thoroughly in FePt grains and boundary areas in EDX mapping in Figure 11 and Figure 12 but more segregated at the grain boundaries in EELS mapping presented in Figure 9. In principle, the EELS mapping data are more accurate for light element N. In Figure 12, the heavy metallic Re appeared in the FePt grains and was accumulated at the FePt(BN, Re, C) and MgO interface, and the Re was overlapped with O and formed ReO2 on the FePt grain surface. It is suggested that the metallic Re will replace part of the Fe in the FePt lattice due to the formation of iron oxide on the film surface and the composition was shifted a little to the Pt-rich FePt(Re) alloy. Furthermore, the hybridization of 3d Fe and 5d Pt(Re) electrons presents high spin–orbital coupling, which reflects high magnetocrystalline anisotropy (K_u_).

## 4. Conclusions

In conclusion, the Re segregant in the FePt film did not improve the ordering degree like Ag but induced extra Fe-Pt(Re) spin–orbital coupling. As a result, the magnetic anisotropic field Hk and Ku can still be high as compared to the reference sample FePt(BN, Ag, C). It is suggested that 5d heavy transition metal segregants such as Re may compensate for the disadvantage of lower ordering degree in the structure, and the lower ordering of the FePt(BN, Re, C) film may be better understood from growth disorientation and defects in between interfaces. Therefore, this study provides the segregant design result for HAMR using FePt media with granular and columnar grains, showing that the 5d heavy transition metal can be used to replace the Ag segregant.

## Figures and Tables

**Table 1:** Magnetic properties of reference FePt(BN, Ag, C) sample and FePt(BN, Re, C) films.

Sample	H_c⊥_ (kOe)	H_c//_ (kOe)	M_s_ (T)	M_r//_/M_r⊥_ (---)	K_u_ (erg/cm^3^)	H_k_ (kOe)	H_N_ (kOe)
FePt(BN, Ag, C)	32	4.5	0.63	0.12	1.74 × 10^7^	69.5	−2.5
FePt(BN, Re, C)	27	2.5	0.55	0.08	1.46 × 10^7^	66.9	−1.8

## References

[1] Hono K., Takahashi Y.K., Varvaro G., Casoli F. (2016). L10 FePt Granular Films for Heat-Assisted Magnetic Recording Media. Ultrahigh Density Magnetic Recording.

[2] Weller D., Parker G., Mosendz O., Lyberatos A., Mitin D., Safonova N.Y., Albrecht M. (2016). Review Article: FePt heat-assisted magnetic recording media. J. Vac. Sci. Technol. B.

[3] Weller D., Mosendz O., Parker G., Pisana S., Santos T.S. (2013). L10 FePtX–Y media for heat-assisted magnetic recording. Phys. Status Solidi A.

[4] Futamoto M., Ohtake M. (2017). Development of Media Nanostructure for Perpendicular Magnetic Recording. J. Magn. Soc. Jpn..

[5] Xu C., Zhou B., Du T., Varaprasad B.S.D.C.S., Laughlin D.E., Zhu J. (2022). Understanding the growth of high-aspect-ratio grains in granular L10-FePt thin-film magnetic media. APL Mater..

[6] Zhou B., Laughlin D.E., Zhu J.G. (2021). The utilization of boron nitride (BN) for granular L10-FePt HAMR media fabrication. Appl. Phys. Letter..

[7] Ju G.P., Peng Y.G., Chang E.K.C., Ding Y., Wu A.Q., Zhu X., Kubota Y., Klemmer T.J., Amini H., Gao L. (2015). High-Density Heat-Assisted Magnetic Recording Media and Advanced Characterization—Progress and Challenges. IEEE. Trans. Magn..

[8] Chen S., Xie Q., Zhou C., Zhou J., Deng J., Guo R., Peng Y.G., Ju G., Chen J.S. (2020). Structure, magnetic and thermal properties of FePt-C-BN granular films for heat assisted magnetic recording. J. Phys. D.

[9] Platt C.L., Wierman K.W., Svedberg E.B., Van de Veerdonk R., Howard J.K., Roy A.G., Laughlin D.E. (2002). L–1 0 ordering and microstructure of FePt thin films with Cu, Ag, and Au additive. J. Appl. Phys..

[10] Gilbert D.A., Wang L.W., Klemmer T.J., Thiele J.U., Lai C.H., Liu K. (2013). Tuning magnetic anisotropy in (001) oriented L10 (Fe12xCux)55Pt45 films. Appl. Phys. Lett..

[11] Zhang L., Takahashi Y.K., Perumal A., Hono K. (2010). L10-orderedhighcoercivity(FePt)Ag–C granular thin films for perpendicular recording. J. Magn. Magn. Mater..

[12] Deng J.Y., Dong K.F., Peng Y.G., Ju G.P., Hu J.F., Chow G.M., Chen J.S. (2016). Effect of TiON-MgO intermediate layer on microstructure and magnetic properties of L10 FePt-C-SiO_2_ films. J. Magn. Magn. Mater..

[13] Tsai J.L., Li H.K., Pan Z.Y., Chang Y.S., Chen Y.R., Pi C., Wu Y.T., Chang C.W. (2017). Magnetic Properties and Microstructure of FePt Films with MgTiON Intermediate Layer. IEEE Trans. Magn..

[14] Tsai J.L., Tzeng J.L., Hu K.C., Li H.K., Pan Z.Y., Chang Y.S., Liao C.C. (2017). Microstructure and magnetic properties of FePt film with combined MoC/(Mg–X)O (X = Cu, Ni, Co) intermediate layers. J. Magn. Magn. Mater..

[15] Sepehri-Amin H., Nagano M., Seki T., Ho H., Tripathy D., Pirzada S., Srinivasan K., Yuan H., Dorsey P., Ajan A. (2018). Microstructure and magnetic properties of FePt-(C, SiO_2_) granular films deposited on MgO, MgTiO, and MgTiON underlayers. Scr. Mater..

[16] Hung S.H., McKenna K. (2017). First-principles prediction of the morphology of L10 FePt nanoparticles supported on Mg(Ti)O for heat-assisted magnetic recording applications. Phys. Rev. Mater..

[17] Chen J.S., Lim B.C., Ding Y.F., Chow G.M. (2006). Low-temperature deposition of L10 FePt films for ultra-high density magnetic recording. J. Magn. Magn. Mater..

[18] Li H.H., Hu J.F., Ju G., Chow G.M., Chen J.S. (2011). Effects of CrRu–SiOx underlayer with MgO intermediate layer on the microstructure and magnetic properties of FePt–C thin film. J. Appl. Phys..

[19] Tsai J.L., Weng S.M., Dai C., Chen J.Y., Huang L.C., Hsu T.W. (2020). Surface modification of FePt(Ag, C) granular film by ultrathin B4C capping layer. Appl. Surf. Sci..

[20] Yu D., Zhou X., Zhang T., Zhong H., Fu Y., Cui W., Wang Q. (2017). Effects of B4C Addition on the Microstructure and Magnetic Properties of FePt-C Granular Thin Films for Perpendicular Magnetic Recording. IEEE Trans. Magn..

[21] Gbadamasi S., Mohiuddin M., Krishnamurthi V., Verma R., Khan M.W., Pathak S., Kalantar-Zadeh K., Mahmood N. (2021). Interface chemistry of two-dimensional heterostructures–fundamentals to applications. Chem. Soc. Rev..

[22] Kadyrzhanov K.K., Shlimas D.I., Kozlovskiy A.L., Zdorovets M.V. (2020). Research of the shielding effect and radiation resistance of composite CuBi2O4 films as well as their practical applications. J. Mater. Sci. Mater. Electron..

[23] Sharko S.A., Serokurova A.I., Novitskii N.N., Ketsko V.A., Smirnova M.N., Almuqrin A.H., Sayyed M.I., Trukhanov S.V., Trukhanov A.V. (2022). A new approach to the formation of nanosized gold and beryllium films by ion-beam sputtering deposition. Nanomaterials.

[24] Kozlovskiy A.L., Zdorovets M.V. (2019). Synthesis, structural, strength and corrosion properties of thin films of the type CuX (X = Bi, Mg, Ni). J. Mater. Sci.: Mater. Electron..

[25] Zubar T.I., Usovich T.I., Tishkevich D.I., Kanafyev O.D., Fedkin V.A., Kotelnikova A.N., Panasyuk M.I., Kurochka A.S., Nuriev A.V., Idris A.M. (2022). Features of galvanostatic electrodeposition of NiFe films with composition gradient: Influence of substrate characteristics. Nanomaterials.

[26] Kikuchi N., Kitakami O., Okamoto S., Shimada Y., Sakuma A., Otani Y., Fukamichi K. (1999). Influence of 5d transition elements on the magnetocrystalline anisotropy of hcp-Co. J. Phys. Condens. Matter..

[27] Pisana S., Mosendz O., Parker G.J., Reiner J.W., Santos T.S., McCallum A.T., Richter H.J., Weller D. (2013). Effects of grain microstructure on magnetic properties in FePtAg-C media for heat assisted magnetic recording. J. Appl. Phys..

[28] Nemoto H., Takekuma I., Nakagawa H., Ichihara T., Araki R., Hosoe Y. (2008). Designing magnetics of capped perpendicular media with minor-loop analysis. J. Magn. Magn. Mater..

[29] Yang E., Laughlin D.E., Zhu J.G. (2012). Correction of order parameter calculations for FePt perpendicular thin films. IEEE Trans. Magn..

[30] Granz S.D., Kryder M.H. (2012). Granular L10 FePt (001) thin films for heat assisted magnetic recording. J. Magn. Magn. Mater..

[31] Inoue K., Shima H., Fujita A., Ishida K., Oikawa K., Fukamichi K. (2006). Temperature dependence of magnetocrystalline ani-sotropy constants in the single variant state of L10-type FePt bulk single crystal. Appl. Phys. Lett..

[32] Richter H.J., Hellwig O., Florez S., Brombacher C., Albrecht M. (2011). Anisotropy measurements of FePt thin films. J. Appl. Phys..

[33] Tsai J.L., Tseng Y.T., Li C.R., Fu S.C. (2010). Magnetization reversal process in Fe/FePt films. Appl. Phys. Lett..

[34] Tsai J.L., Weng S.M., Dai C., Chen J.Y., Lu X.C., Hsu T.W. (2021). Temperature Dependence and Microstructure Effects on Magnetic Properties of FePt(B, Ag, C) Film. Nanomaterials..

[35] Trukhanov S.V., Trukhanov A.V., Vasiliev A.N., Balagurov A.M., Szymczak H. (2011). Magnetic state of the structural separated anion-deficient La_0.70_Sr_0.30_MnO_2.85_ manganite. J. Exp. Theor. Phys..

[36] Kozlovskiy A., Egizbek K., Zdorovets M.V., Ibragimova M., Shumskaya A., Rogachev A.A., Ignatovich Z.V., Kadyrzhanov K. (2020). Evaluation of the efficiency of detection and capture of manganese in aqueous solutions of FeCeOx nanocomposites doped with Nb_2_O_5_. Sensors.

[37] Yang J., Hou W., Pan R., Zhou M., Zhang S., Zhang Y. (2022). The interfacial electronic engineering in polyhedral MOF derived Co-doped NiSe2 composite for upgrading rate and longevity performance of aqueous energy storage. J. Alloys Compd..

[38] Krishna M.G., Bhattacharya A.K. (2000). Growth of rhenium oxide thin films. Solid State Commun..

[39] Ho H., Zhu J., Kulovits A., Laughlin D.E., Zhu J.G. (2014). Quantitative transmission electron microscopy analysis of multi-variant grains in present L10-FePt based heat assisted magnetic recording media. J. Appl. Phys..

[40] Varaprasad B.S.D.C.S., Takahashi Y.K., Wang J., Ina T., Nakamura T., Ueno W., Nitta K., Uruga T., Hono K. (2014). Mechanism of coercivity enhancement by Ag addition in FePt-C granular films for heat assisted magnetic recording media. Appl. Phys. Lett..

